# Cyclooxygenase enzyme and PGE2 expression in patients with functional and non-functional pituitary adenomas

**DOI:** 10.1186/s12902-020-0515-8

**Published:** 2020-03-14

**Authors:** Nasrin Akbari, Mohammad Ghorbani, Vahid Salimi, Alimohammad Alimohammadi, Mohammad E. Khamseh, Hamideh Akbari, Mitra Nourbakhsh, Alireza Sheikhi, S. Fahimeh Taghavi, Masoumeh Tavakoli-Yaraki

**Affiliations:** 1 Department of Biochemistry, School of Medicine, Iran University of Medical Sciences, P.O. Box: 1449614535, Tehran, Iran; 2 Division of Vascular and Endovascular Neurosurgery, Firoozgar Hospital, Iran University of Medical Sciences, Tehran, Iran; 3 Department of Virology, School of Public Health, Tehran University of Medical Sciences, Tehran, Iran; 4 Iranian Legal Medicine Organizations, Tehran, Iran; 5 Endocrine Research Center, Institute of Endocrinology and Metabolism, Iran University of Medical Sciences (IUMS), Tehran, Iran; 6 Clinical Research Development Unit (CRDU), Sayad Shirazi Hospital, Golestan University of Medical Sciences, Gorgan, Iran

**Keywords:** Pituitary adenoma, Cyclooxygenase-1, Cyclooxygenase-2, PGE2

## Abstract

**Background:**

Pituitary adenomas as multifactorial intracranial neoplasms impose a massive burden of morbidity on patients and characterizing the molecular mechanism underlying their pathogenesis has received considerable attention. Despite the appealing role of cyclooxygenase enzymes and their bioactive lipid products in cancer pathogenesis, their relevance to pituitary adenoma pathogenesis is debated and yet to be determined. Thus, the current study perused this relevance.

**Methods:**

The expression level of the isoforms of cyclooxygenase (COX-1 and COX-2) was evaluated in hormone-secreting and in-active pituitary adenoma tumors and normal pituitary tissues through Real-Time PCR. The level of PGE2, as the main product of enzymes, was assessed using enzyme immunoassay kits in patients and healthy subjects.

**Results:**

The results of the current study demonstrated that COX-1 and COX-2 expression levels were increased in pituitary tumors including non-functional pituitary adenoma (NFPA), acromegaly, Cushing’s disease and prolactinoma compared with normal pituitary tissues. A significant expression level of COX-2 was observed in NFPA compared with the other pituitary tumors. Furthermore, the COX-2 expression level was significantly increased in macroadenoma and invasive tumors. The level of PGE2 was consistent with COX enzymes enhanced in pituitary adenoma tumors compared with healthy pituitary tissue. A significant elevation in the PGE2 level was detected in NFPA compared with hormone-secreting pituitary tumors. Additionally, the PGE2 level was increased in macroadenoma compared with microadenoma and in invasive compared with non-invasive pituitary tumors. The diagnostic values of cyclooxygenase isoforms and PGE2 were considerable between patients and healthy groups; however, COX-2 revealed more value in distinguishing endocrinologically active and non-active pituitary tumors.

**Conclusions:**

Data from the current study provides expression patterns of COX-1, COX-2 and PGE2 in prevalent pituitary tumors and their association with patients’ clinical features which may open up new molecular targets for early diagnosis/follow up of pituitary tumor growth.

## Background

The molecular mechanism by which tumor cells are grown, proliferated, developed and metastasized is under extensive investigation. The inter-tumor heterogeneity [1], microenvironment of tumor cells [2], presence of cancer-stem cell populations [3] and inflammatory mediators [4] are the most recently determined cellular events by which tumor cell fate is influenced. Among the molecular pathways related to inflammation, the enzymes of the prostaglandin pathway are well characterized as inflammatory mediators which are implicated in the pathogenesis of cancer and other diseases [5, 6]. Cyclooxygenases are the peroxidases responsible for converting arachidonic acid to prostaglandins that can be released from cells and activated specified receptors, triggering cellular signaling pathways mediating fever, vasodilation, platelet aggregation, pain and inflammation [4, 7, 8]. The constitutively expressed cyclooxygenase isoform, the so-called cycloxygensae-1 (COX-1) is located in chromosome 9 and is involved in cell proliferation, angiogenesis and tissue hemostasis [7]. The inducible isoform of cyclooxygenase, namely- cyclooxygenase-2 (COX-2), is located in chromosome 1 and stimulated by growth factors and cytokines and contributed in inflammatory responses [9]. The overexpression of COX-2 has been reported in several types of tumors, including breast, pancreatic, lung, colorectal, leukemia and lymphoma tumors [4]. Accumulated evidences has revealed that COX-2 is involved in tumor cell proliferation [10], invasion [11], angiogenesis [12], apoptosis [13], drug resistance [14] and immune evasion [15]. The prostaglandin E2 (PGE2) as the main product of COX-2 mediates its effects in cancer cell proliferation, invasion and death through activation of the PGE_2_ receptor (EP) and subsequent induction of cAMP and protein kinase K [16]. Additionally, PGE2 is able to suppress apoptosis by attenuating the level of pro-apoptotic mediators in colorectal cancer [17]. Activation of the Src kinase and the subsequent phosphorylation of STAT3, which positively regulates cell cycle effectors, are supposed to be mediated by PGE2 in lung cancer [18]. Also, the promoting impact of PGE2 on VEGF expression emphasizes its role in angiogenesis and facilitation of tumor invasion [19–21]. Despite the fact that COX isoforms share structural and enzymatic similarities, different regulatory approaches, tissue distribution and the subsequent activities are believed to affect their role in cell function [7]. The accurate role of COX-1 in cancer pathogenesis is debated and less considered, while elevated levels of COX-1 have been detected in some types of cancers. Accordingly, the over-expression of COX-1 has been correlated with the poor prognosis of renal cell carcinoma [22]. The up-regulation of COX-1 has been revealed in the cytoplasm of neoplastic cells in head and neck cancer [23]. It seems that both isoforms influence cancer pathogenesis coordinately; however, this premise should be verified with further evidences. The pituitary gland function is linked to the normal physiology of the human body and tightly related to the metabolism, reproduction, stress controlling and immune responses. The dynamic adjustment of pituitary gland cells to meet the hormonal requirement of the body during growth and maturation makes this master gland more appealing [24]. The heterogeneous group of tumors arises from the pituitary gland which accounts for approximately 20% of intracranial neoplasms. Pituitary adenomas are classified mainly based on their ability to change hormonal level, tumor size and invasiveness [25]. As hormone-secreting pituitary adenomas, prolactinoma (excess of prolactin), Cushing’s disease (excess of ACTH) and acromegaly (excess of growth hormone) are the most prevalent types, although non-functional pituitary adenomas which lack hormone overproduction, affect a large number of patients worldwide, and their diagnosis is more challenging [26]. Characterization of the pathophysiology of pituitary adenomas might pave the way for the early diagnosis and follow up of pituitary adenomas. Despite few observations regarding the over expression of COX-2 in pituitary carcinomas, the expression patterns of both COX isoforms and PGE2 in different types of pituitary adenomas and their correlations with tumor types, size and behavior have yet to be determined; thus, they were considered and investigated in the current study.

## Methods

### Patients and sample collection

The total number of 91 pituitary samples including 71 pituitary adenomas and 20 cadaveric healthy pituitary tissues were enrolled in the current study with local ethical approval and informed consent which was ethically approved by the ethics committee of vice president of research in Iran University of Medical Sciences. The tumor and normal pituitary sample collection was followed as the method of Barooni et al. [27]. Notably, to avoid the possible effect of pituitary adenomas medical treatments on the COX expression and PGE2 level assessment, only the patients who received no treatments prior to surgery were selected and enrolled in this study. The clinic- pathological features of patients with pituitary adenoma are summarized in Table 1. Additionally, invasive pituitary tumors were classified in terms of the Knosp classification system in which tumor extension to the adjacent sphenoid sinus and cavernous sinus is defined as invasive tumor. Also, in our study the tumor tissues greater than 10 mm in size were considered as macroadenomas and tumors less than 10 mm in size were defined as microadenomas. The patient’s biochemical features and their hormone levels were assessed and summarized in Table 2.

**Table 1:** The clinic- pathological features of patients with pituitary adenoma

Parameter	Groups	Pituitary adenoma (*n* = 71)	Non-Functional pituitary adenoma (*n* = 30)	Functional pituitary adenoma (*n* = 41)	Acromegaly (*n* = 20)	Cushing (*n* = 13)	Prolactinoma (*n* = 8)
Age (Years)	20–40	34(47.88%)	13(43.33%)	21(51.21%)	10(50%)	7(53.84%)	4(50%)
	40–60	22(30.98%)	9(30%)	13(31.70%)	6(30%)	5(38.46%)	2(25%)
	60≤	15(21.12%)	8(26.66%)	7(17.07%)	4(20%)	1(7.69%)	2(25%)
Gender	Female	34(47.88%)	12(40%)	22(53.65%)	11(55%)	8(61.53%)	3(37.5%)
	Male	37(52.11%)	18(60%)	19(46.34%)	9(45%)	5(38.46%)	5(62.5%)
Tumor invasiveness	Invasive	33(46.47%)	13(43.33%)	20(48.78%)	10(50%)	4(30.76%)	6(75%)
	Non- Invasive	38(53.52%)	17(56.66%)	21(51.21%)	10(50%)	9(69.23%)	2(25%)
Tumor size	Micro-Adenoma	16(22.53%)	0	16(39.02%)	8(40%)	8(61.53%)	0
	Macro-Adenoma	55(77.46%)	30(100%)	25(60.97%)	12(60%)	5(38.46%)	8(100%)

**Table 2:** The biochemical features of patients with pituitary adenoma

Demographic features	NFPA	Acromegaly	Cushing	Prolactinoma
Age	55.12 ± 5.12	45.23 ± 3.2	35.7 ± 2.3	44 ± 9.44
Tumor size	29.02 ± 3.2	16.02 ± 1.08	9.31 ± 1.42	28.7 ± 2.14
Blood Sugar (100-145 mg/dl)	156 ± 7.7	154.2 ± 10.2	215 ± 23.20	118 ± 21.43
Na (135–145 mmol/L)	143.2 ± 1.99	143.1 ± 1.15	152.7 ± 5.2	141 ± 3.44
K (3.5–5.5 mmol/L)	3.9 ± 0.36	3.9 ± 0.13	3.7 ± 0.3	3.45 ± 0.32
Urea (14-43 mg/dl)	43.91 ± 6.7	41.6 ± 3.7	45.60 ± 8.5	32.15 ± 3.6
WBC (4–10*1000/mm^3^)	9.4 ± 0.5	8.01 ± 0.41	10.4 ± 0.7	9.2 ± 0.3
Prolactin				
Men: 87–392 mIU/L	309.1 ± 100	719 ± 41	227 ± 23	38,920 ± 20
Women productive age: 132–498 mIU/L Post menopause: 90–392 mIU/L	701.5 ± 25	945 ± 52	325 ± 42	44,684 ± 15
Growth Hormone				
Women:0.126–9.88 ng/ml	0.2 ± 0.05	12.03 ± 3.25	0.1 ± 0.01	0.06 ± 0.03
Men:0.03–2.47 ng/ml	0.25 ± 0.03	7.62 ± 1.4	0.32 ± 0.04	0.15 ± 0.02
ACTH (7.2–63.3 pg/ml)	24.15 ± 6	41.47 ± 6.13	192.7 ± 52.14	29.7 ± 11
Cortisol (morning)6.2–19.4 μg/dl	11.22 ± 3.77	20.11 ± 4.5	34.43 ± 8.2	7.22 ± 1.2
Testosterone				
Men:2.5–10 ng/ml	2.3 ± 1.7	1.02 ± 0.06	1.4 ± 1.02	1.2 ± 0.2
women:0.2–0.95 ng/ml	0.23 ± 0.05	0.24 ± 0.16	0.09 ± 0.5	0.11 ± 0.6
IGF-1 53-234 ng/ml	172.8 ± 22.4	662.9 ± 52.14	236 ± 22	95.4 ± 3.4

### RNA extraction, cDNA synthesis, real-time PCR

The expression levels of COX isoforms were measured using specific primers via Real-Time PCR based on the methods and materials described previously [27]. The sequence and characters of primers are listed in Table 3. The beta-actin expression level, as a housekeeping target gene, was measured to normalize the COX isoforms expression level. The beta-actin is considered as a housekeeping gene with the most constant level in the brain and pituitary tissues. To evaluate the accuracy of beta-actin, the serial dilution of cDNA at the concentrations of 0.01, 0.1, 1, 10, and 100 ng / μl were prepared, and the standard curve was constructed with the logarithm of the initial copy number of the standards plotted along with the x-axis, and their respective CT values were plotted along with the y-axis. The low CT values as well as the standard efficiency confirmed the beta-actin as a suitable housekeeping gene for our assay.

**Table 3:** Primers used for qRT-PCR assessment of gene expressions

Gene	Primers	Primer sequence	Tm
*Cox-1*	Forward	5-GGTAACTGCTTAGGACCAGT-3	58
	Reverse	5-ACTGTTCTCCGTACCTTCAC-3	
*Cox-2*	Forward	5-TGTCATTCCAGAGTGCTGAG-3	58
	Reverse	5-GGCTAACAAGGAGTTCAGCA-3	
Beta-Actin	Forward	5-GAT CTC CTT CTG CAT CCT GT-3′	57
	Reverse	5′-TGG GCA TCC ACG AAA CTA C- 3’	

### ELISA measurement of PGE2 level

The level of PGE2 as a main product of COX isoforms was assessed in tissue homogenates of tumor and normal pituitary tissues using an enzyme immunoassay kit (Cayman Chemicals, USA) in terms of the manufacturer’s protocol. The kit sensitivity was 15 pg/ml and the wavelength of 405 nm was applied for the absorbance detection using microplate reader.

### Statistical analysis

In order to analyze the gene expression levels, the comparative CT (2^-ΔCt^) method was applied. The In-dependant t-test was used to analyse the differences of COX mRNA expression and PEG2 level between the main groups. In-dependant t-test compares the mean between two unrelated groups on the same variable. Also, the one-way analysis of variance (ANOVA) was used to determine the statistically significant differences between the means of more than two unrelated groups; for example, COX-1 expression between functional pituitary adenoma subtypes. To delineate the possible correlations among patient’s clinic pathological features and the assayed factors, the Pearson correlation coefficient test was applied to indicate the possible relationship and association between variables (Tables 4, 5). The diagnostic values of COX-1, COX-2 and PEG2 were assessed using the receiver operating characteristic (ROC) curves. The statistical calculations were calculated using the Graph Pad Prism version 6 (Graph Pad Software, San Diego California) and Statistical Package for Social Science (SPSS v.20) with consideration of *p*-values < 0.05 to clarify the significant difference between tested groups.

**Table 4:** COX-1 Correlation with patient’s clinic pathological features

Parameter	Groups	Non-Functional pituitary adenoma *(P value)*	Acromegaly *(P value)*	Cushing *(P value)*	Prolactinoma *(P value)*
Age (Years)	20–40	0.8	0.86	0.86	0
	40–60	0.92	0.78	0.62	
	60≤				
Gender	Female	0.18	0.35	0.05	0.73
	Male				
Tumor invasiveness	Invasive	0.19	0.54	0.18	0
	Non- Invasive				
Tumor size	Micro-Adenoma	0	0.62	0.65	0
	Macro-adenoma

**Table 5:** COX-2 Correlation with patient’s clinic pathological features

Parameter	Groups	Non-Functional pituitary adenoma *(P value)*	Acromegaly *(P value)*	Cushing *(P value)*	Prolactinoma *(P value)*
Age (Years)	20–40	0.88	0.9	0.4	0
	40–60	0.56	0.17	0.61	
	60≤				
Gender	Female	0.29	0.76	0.04	0.057
	Male				
Tumor invasiveness	Invasive	0.05	0.84	0.21	0
	Non- Invasive				
Tumor size	Micro-Adenoma	0	0.54	0.07	0
	Macro-adenoma				

## Results

### The COX-1 expression level enhanced in tumor tissues of different pituitary adenomas

**Fig. 1:**
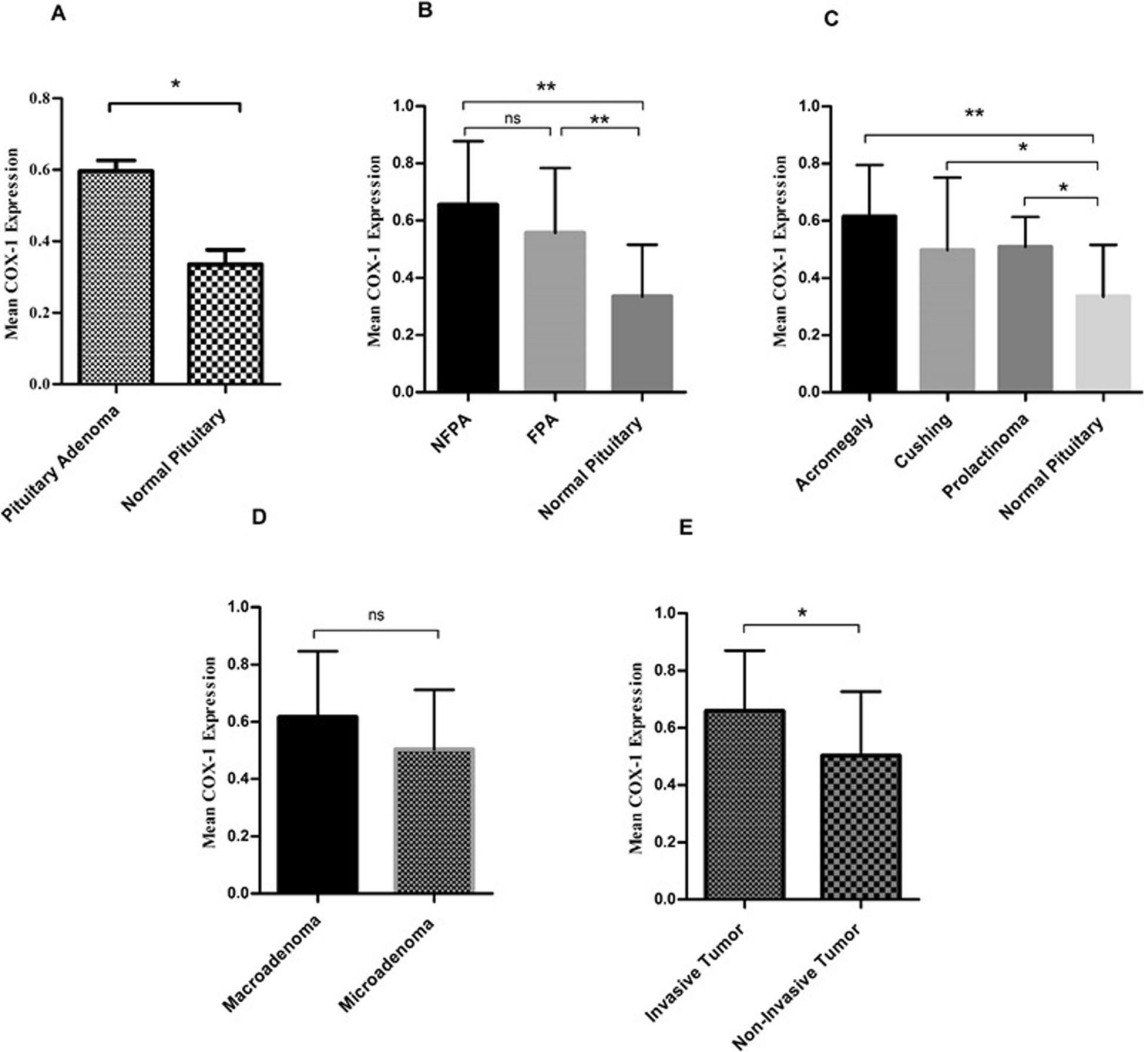
COX-1 expression level in pituitary adenomas**.** The expression level of COX-1 was evaluated in tumor and normal tissues of pituitary with different adenoma types. The COX-1 expression level was increased in pituitary adenoma (**a**), non-functional pituitary adenoma (**b**), functional pituitary adenoma subtypes (**c**), macroadenoma (**d**) and invasive (**e**) tumor tissues. The Statistical differences between groups is shown as asterisk (* = *P* < 0.05, ** = *P* < 0.01)

In order to determine the COX-1 expression status in pituitary adenomas, the COX-1 gene expression level was measured in 91 pituitary tissues including 71 pituitary adenoma tumor and 20 normal pituitary tissues using Real-Time PCR. Based on the obtained data, the COX-1 expression level was elevated in tumor tissues compared to normal pituitary tissue (*P* = 0.04) (Fig. 1a). Following the analysis, the mean and the standard error mean (SEM) of COX-1 mRNA level were 0.59 ± 0.02 and 0.33 ± 0.04 in patients and control group, respectively, which were calculated via 2^-ΔCt^ method. Accordingly, the approximately 1.78 fold increase in the level of COX-1 was observed in tumor tissues compared with healthy pituitary tissue. Moreover, to address the status of COX-1 expression level in different types of pituitary adenomas, the tumor tissues of FPA and NFPA were included in the study and it has been shown that COX-1 expression level increased in both FPA and NFPA compared with normal pituitary tissue; however, no significant difference was observed between FPA and NFPA groups (Fig. 1b). Accordingly, the mean and the SEM of COX-1 mRNA level were 0.65 ± 0.042, 0.55 ± 0.03, and 0.33 ± 0.04 in NFPAs, FPAs, and normal tissues, respectively. Also, the approximate 1.96 and 1.66 fold increase were observed in the COX-1 expression level in NFPAs and FPAs compared to normal tissues, respectively. Among the prevalent types of FPAs, it was observed that higher level of COX-1 was expressed in tumor tissues of acromegaly (0.61 ± 0.048), Cushing (0.49 ± 0.067), and prolactinoma (0.50 ± 0.037) groups compared with normal tissue; however, the differences between FPAs group were not remarkable (Fig. 1c). Also, the approximate 1.84, 1.48, and 1.51 fold increase were observed in the COX-1 expression levels in acromegaly, Cushing, and prolactinoma comparing to normal tissues, respectively. Tumor size and invasiveness are accounted as sever features of tumors in pituitary adenomas; therefore, these two characters were included in the analysis. As shown in Tables 1, 22.53% of pituitary tumors in our study were categorized as microadenomas and 77.46% were macroadenomas. Based on the data, no significant difference was observed regarding the expression levels of COX-1 in macro- and micro-adenomas (Fig. 1d). Additionally, 46.47% of tumors included in the study were invasive and 53.52% of them were non-invasive (Table 1), which as a point of COX-1 expression, invasive tumors (0.65 ± 0.035) expressed higher level of COX-1 compared with the non-invasive tumors (0.50 ± 0.045), (*P* = 0.015) and this revealed the approximate 1.3 fold increase (Fig. 1e). The correlation of patients’ clinic pathological features with COX-1 expression level was evaluated, and despite the significant correlation of COX-1 with gender in Cushing patients, no significant correlation was observed between the patients’ features and COX-1expression, which is summarized in Table 4.

### COX-2 revealed higher level of expression in tumor tissues of different pituitary adenomas comparing to healthy pituitary tissues

**Fig. 2:**
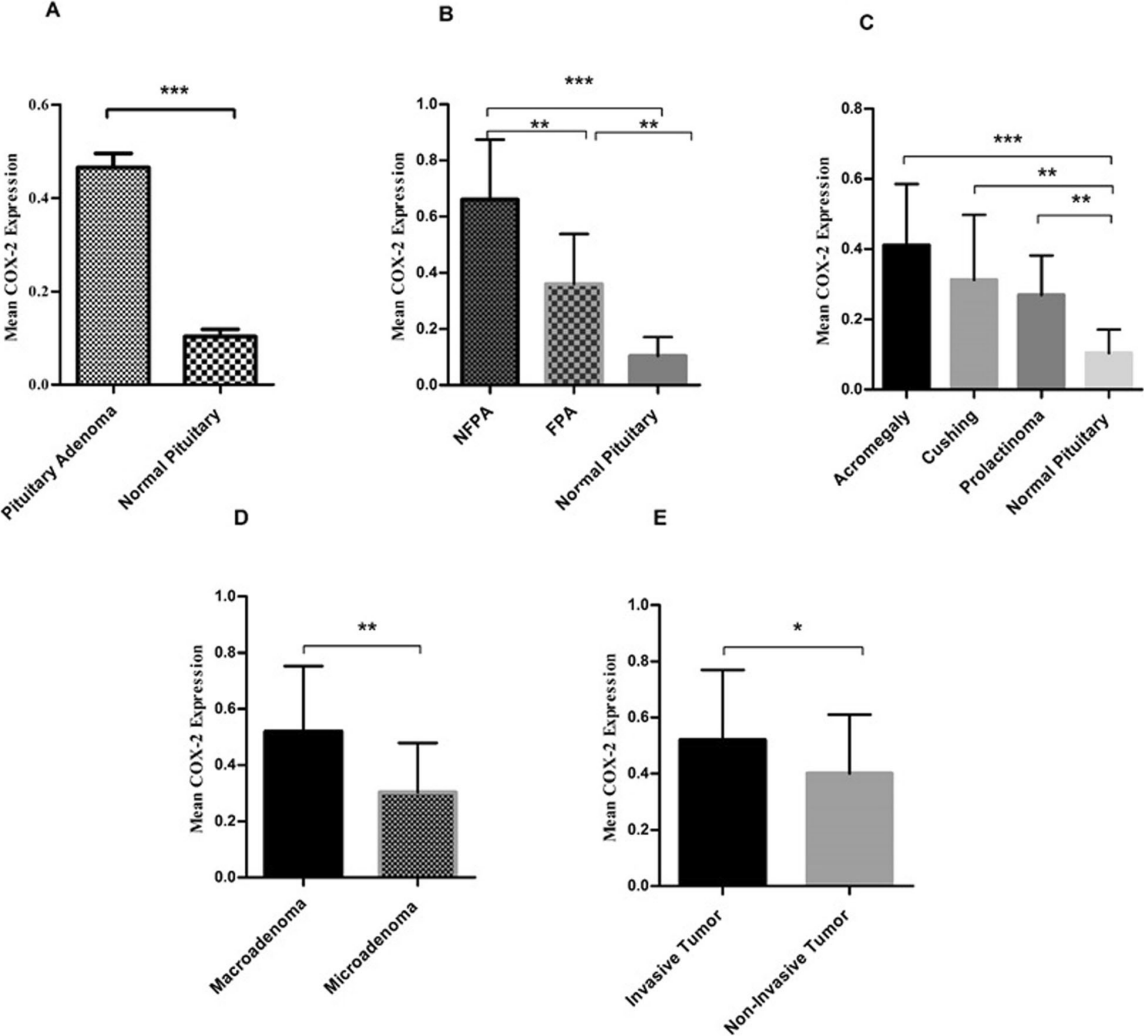
COX-2 expression level in pituitary adenomas. The expression level of COX-2 was evaluated in tumor and normal tissues of pituitary with different adenoma types. The COX-2 expression level was increased in pituitary adenoma (**a**), non-functional pituitary adenoma (**b**), functional pituitary adenoma subtypes (**c**), macroadenoma (**d**) and invasive (**e**) tumor tissues. The Statistical differences between groups is shown as asterisk (* = *P* < 0.05, ** = *P* < 0.01,*** = *P* < 0.001)

Multiple lines of evidence demonstrated the involvement of inducible isoform of cyclooxygenase (COX-2) in tumor genesis although, its status in pituitary adenoma pathogenesis has yet to be determined [4]. Accordingly, the COX-2 gene expression was assessed in different types of pituitary adenomas. As it is reveled in Fig. 2a pituitary tumor tissues expressed higher level of COX-2 compared to normal pituitary tissue (*P* = 0.0001) with the mean and the standard error mean (SEM) of 0.4651 ± 0.02 and 0.1034 ± 0.01 for patients and control groups, respectively. The analysis which were calculated by 2^-ΔCt^ method revealing the approximately 4.4 fold increase in the level of COX-2 in tumor tissues compared to healthy pituitary. Based on the obtained data, the expression level of COX-2 was significantly elevated in NFPAs compared with FPAs (*P* = 0.001). The elevated level of COX-2 in both FPA and NFPA groups compared with normal pituitary were detected (Fig. 2b). Accordingly, the mean and the SEM of COX-2 mRNA level were 0.66 ± 0.04, 0.365 ± 0.027, and 0.1034 ± 0.01 in NFPAs, FPAs, and normal tissues, respectively. Also, the approximate 6.6 and 3.6 fold increase were observed in the COX-2 expression level in NFPAs and FPAs compared to normal tissues, respectively Among the FPAs, tumor tissues of the patients with acromegaly (0.41 ± 0.039) revealed higher level of COX-2 compared to Cushing (0.31 ± 0.053) and prolactinoma (0.26 ± 0.039) patients; however, the observed differences were not statistically significant between groups Also, the approximate 4.1, 3.1, and 2.6 fold increase were observed in the COX-2 expression levels in acromegaly, Cushing, and prolactinoma comparing to normal tissues, respectively (Fig. 2c), interestingly, the macroadeomas tumor tissues (0.52 ± 0.036) expressed more COX-2 compared to microadenomas (0.30 ± 0.043) (*P* = 0.01) and this revealed the approximate 1.73 fold increase (Fig. 2d). Additionally, in terms of invasive tumors the expression level of COX-2 was significantly increased compared to the non-invasive tumors (*P* = 0.04) which were (0.52 ± 0.042) and (0.40 ± 0.039), respectively. (Fig. 2e). It indicated the approximate 1.3 fold increase in the COX-2 expression level in invasive and non-invasive pituitary adenomas. Regarding the correlation of patient’s clinic pathological features with COX-2 expression which is summarized in Table 5, the COX-2 expression seemed to be correlated with gender in Cushing and prolactinoma, although it was not observed for the other types of pituitary adenomas. In addition, the tumor invasiveness was correlated with COX-2 expression in NFPAs which was not observed for the other groups.

### The elevation of PGE2 in pituitary adenomas

**Fig. 3:**
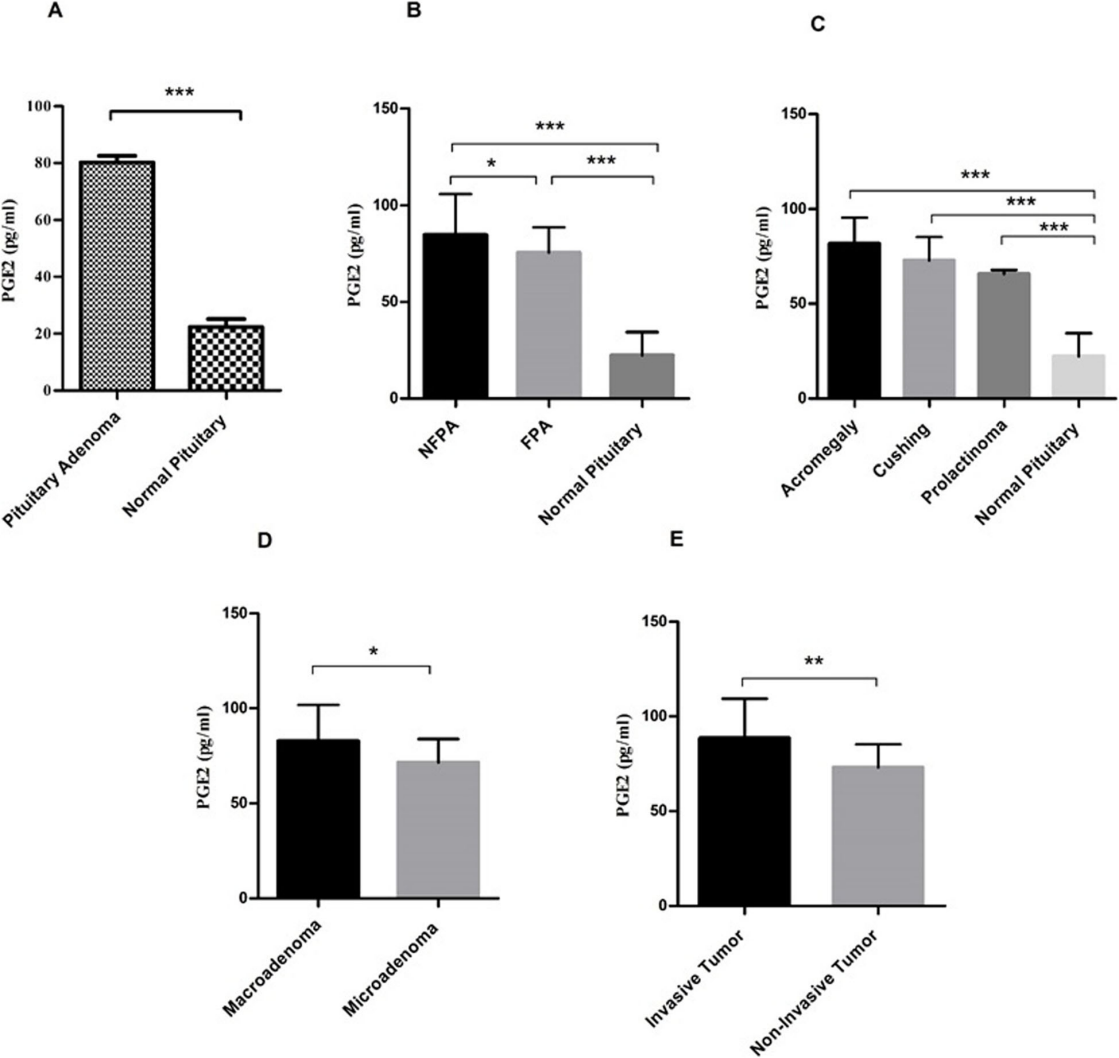
PGE2 level in pituitary adenomas. The level of PGE2 was evaluated in tumor and normal tissues of pituitary with different adenoma types. The PGE2 level was increased in pituitary adenoma (**a**), non-functional pituitary adenoma (**b**), functional pituitary adenoma subtypes (**c**), macroadenoma (**d**) and invasive (**e**) tumor tissues. The Statistical differences between groups is shown as asterisk (* = *P* < 0.05, ** = *P* < 0.01, *** = *P* < 0.001)

The PGE2 exert positive role in cell proliferation, angiogenesis and inflammation, and its synthesis is tightly influenced by COX isoforms [28]. The assessment of this bioactive lipid in tumor and healthy tissues of pituitary in the current study showed the higher level of PGE2 in pituitary tumors compared with normal tissue (*P* = 0.0001) (Fig. 3a). Following the analysis, the mean and the standard error mean (SEM) of PGE2 level were 80.21 ± 2.3 and 22.32 ± 2.7 in patients and control group, respectively, which indicates the 3.59 fold increase in the level of PGE2 patients compared to the healthy individuals. Notably, the PGE2 level was significantly increased in NFPAs (84.61 ± 3.7) compared to FPAs (75.38 ± 2.4) (*P* = 0. 04) (Fig. 3b). Also, the approximate 3.7 and 3.3 fold increase were observed in the PGE2 level in NFPAs and FPAs compared to normal group, respectively. Among the FPAs, the level of PGE2 enhanced in all types of pituitary tumors (Acromegaly (81.68 ± 3.6), Cushing (72.81 ± 24.3) and Prolactinoma (65.70 ± 1.3) compared to the healthy group; however; no remarkable difference was observed between FPAs subtypes (Fig. 3c). Also, the approximate 3.65, 3.26, and 2.9 fold increase were observed in the PGE2 levels in acromegaly, Cushing, and prolactinoma comparing to normal group, respectively The higher level of PGE2 in macroadenoma (82.79 ± 2.7) tumor tissues was observed compared to the microadenoma tissues (71.59 ± 3.2) indicating the 1.15 fold increase (*P* = 0.04) (Fig. 3d), also, the level of PGE2 was elevated in invasive tumors (88.60 ± 3.9) compared to non-invasive tumors (73.11 ± 2.1) revealing the 1.21 fold increase (*P* = 0.001) (Fig. 3e).

### The diagnostic value of COX isoforms and PGE2 in pituitary adenomas

**Fig. 4:**
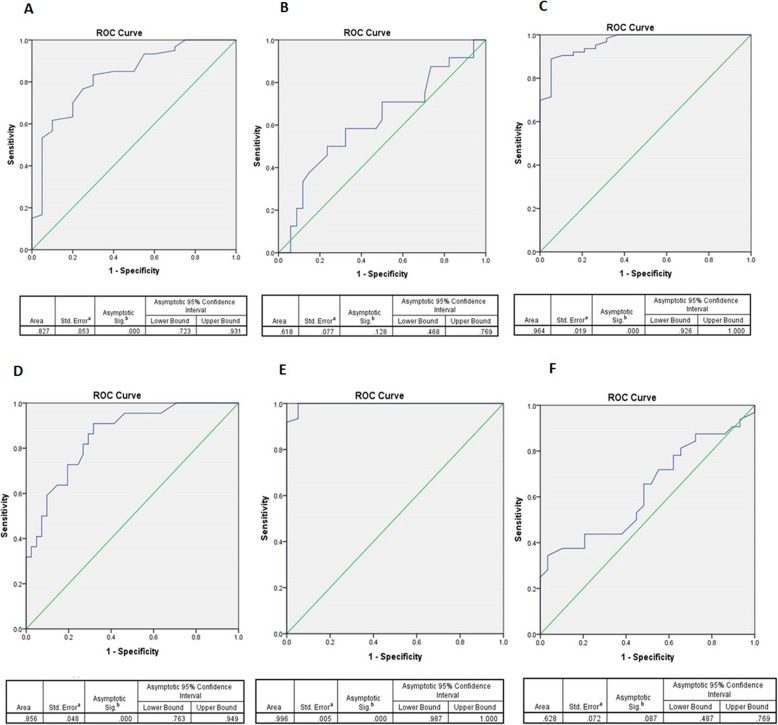
COX-1, COX-2 and PGE2 ROC curve. The COX-1, COX-2 and PGE2 ROC Curve, area under the curve, 95% confidence interval, P value are shown for COX-1 in pituitary patients /healthy tissues (**a**), COX-1 in NFPAs/FPAs (**b**), COX-2 in pituitary patients /healthy tissues (**c**), COX-2 in NFPAs/FPAs (**d**), PGE2 in pituitary patients /healthy tissues (**e**), PGE2 in NFPAs/FPAs (**f**)

The diagnostic value of the COX-1, COX-2 and PGE2 were evaluated between examined groups using ROC curves. The diagnostic values are indicated by area under the curve (AUC) and the cutoff value for each parameter. As it is indicated in Fig. 4a, the COX-1 expression between pituitary tumor and normal tissue group showed the AUC level of 0.827 (95% CI, 0.723–0.931, *P* = 0.0001) and 0.43 as cut off value with 76% sensitivity and 75% specificity based on Youden Index. However; the AUC level of 0.618 (95% CI, 0.468–0.769, *P* = 0.128) and the cut of value of 0.51 (70% sensitivity and 50% specificity) was determined for COX-1 expression diagnostic value between NFPAs and FPAs (Fig. 4b). Notably, the COX-2 gene expression between pituitary tumor and normal tissue group showed the AUC level of 0.964 (95% CI, 0.926–1, *P* = 0.0001) and 0.18 as cut off with 90% sensitivity and specificity (Fig. 4c) while its expression between NFPAs and FPAs group showed the AUC level of 0.856 (95% CI, 0.763–0.949, *P* = 0.0001) and cut off value of 0.42 (86% sensitivity, 71% specificity) (Fig. 4d). The PGE2 revealed the diagnostic value between pituitary tumor and normal tissue group as the AUC level of 0.996 (95% CI, 0.987–1, *P* = 0.0001) and cut off value of 49.8 with 100% sensitivity and specificity (Fig. 4e). However; the AUC level of 0.628 (95% CI, 0.487–0.769, *P* = 0.087) and cut off value of 77.8 (50% sensitivity and 48.9% specificity) was determined for PGE2 level between NFPAs and FPAs group (Fig. 4f). Based on the results, the COX isoforms and PGE2 level can significantly distinguish pituitary tumors versus pituitary normal tissues, however; the COX-2 level showed better diagnostic value between NFPAs and FPAs group.

## Discussion

Pituitary adenomas account for approximately 15% of intracranial neoplasms and impose a devastating burden of morbidity on patients, which arises from the multifunctional role of the pituitary gland in the body [29]. These heterogeneous types of tumors might be endocrinologically active or in-active, the underlying molecular mechanism involving tumor pathogenesis has yet to be characterized. The COX pathway holds great promise as an effective cellular pathway for triggering tumor initiation, progression and apoptosis, and multiple bodies of evidences suggest the over-expression of COX isoforms in several types of tumors, including breast, pancreatic, lung, colorectal, leukemia, lymphoma, renal cell carcinoma and head and neck cancers [4, 22, 23]. Selective COX inhibitors are able to manipulate the fate of cancer cells as well as cell proliferation, invasion, angiogenesis, death and the immune system response, which together reinforce the impact of the COX pathway in cancer pathogenesis [7]. Due to a lack of data regarding the status of COX isoforms in pituitary adenomas, the expression profile of COX isoforms and PGE2 were perused in the current study. The higher expression levels of both COX isoforms were observed in pituitary adenomas compared with normal pituitary tissue; however, the fold change in COX-2 expression level was higher than that of COX-1 in tumors compared with healthy tissue. No similar studies were found to have evaluated the status of COX-1 expression in human pituitary adenoma; however, an elevated level of COX-2 expression was reported in pituitary carcinomas and oncocytic and non-oncocytic null-cell adenomas, although the methodology of that study differed from that of the current study [30, 31]. Onguru et al., measured the protein level of COX-2 by immunohistochemistry, while the current study, assessed the mRNA levels of both COX isoforms, and, in order to delineate whether COX mRNA expression conveys protein expression, assessed the level of PGE2 as an active product of the COX-2 enzyme. The consistency of PGE2 over- production with COX-2 over- expression emphasized the existence of active COX-2 proteins and enzymes in pituitary tumors. The up-regulation of the COX-1 enzyme was detected in some types of tumors, including renal cell carcinoma and head and neck cancers, and the current data indicates a pro-tumor genesis effect of COX-1 in pituitary adenomas. The coordinate expression pattern of COX-1 and COX-2 in pituitary adenomas in the current study indicates the propelling role of both isoforms in pituitary adenoma pathogenesis, although COX-2 distinguished tumors tissues from healthy pituitary tissues more accurately and significantly compared with COX-1. Additionally, COX-1 expression levels were enhanced in invasive tumors, while no significant differences were observed among different types of pituitary tumors or in micro-and macro-adenomas. Based on the data in the current study, COX-2 levels were significantly higher in NFPAs than in FPAs with a significant diagnostic value for distinguishing these two groups. In support of this, Sokołowski et al., revealed that COX-2 was more expressed in non-secreting hormone pituitary adenomas, while prolactinoma patients showed the lowest expression levels of the COX-2 enzyme [32]. In the current study, no difference among the FPAs subtypes as a point of COX-2 expression was observed, although acromegaly tumor tissue and prolactinoma revealed the highest and lowest levels of COX-2 in endocrinologically active pituitary tumors, respectively. This result is in line with previously reported evidence [31, 32]. Apparently, the observed level of COX-2 expression might be affected by the number of acromegaly and prolactinoma participants in the current study, which should be refined by further studies. Interestingly, the up-regulation of COX-2 was accompanied by STAT3 phosphorylation and facilitation of epithelial-mesenchymal transition (EMT), which resulted in osteosarcoma metastasis and invasion [33]. Moreover, inhibition of COX-2 caused attenuated migration and invasion of radiation-resistant lung cancer cells through the involvement of specificity protein 1 (Sp1) [34]. Notably, COX-2 induced HIF2α level and activity and HIF2α nuclear translocation through the MAPK pathway, which consequently enhanced angiogenesis and proliferative mediators in hepatocellular carcinoma [35]. In support of this, the overexpression of COX-2 in invasive pituitary tumors and macroadenomas in the current study might emphasize the accelerating role of COX-2 in inducing higher rates of cell proliferation in pituitary adenomas. The mechanism by which COX-2 mediates pituitary adenoma invasion cannot be concluded from the current results, and further mechanistic studies are required to characterize the exact role of COX-2 in this regard. PGE2 accounts as the main product of COX-2 is involved in various cellular functions including cancer cell proliferation, invasion, and death through the activation of EP receptors and subsequent induction of cAMP and protein kinase K [16]. PGE2 might affect cancer pathogenesis by different mechanisms. For example, it has been reported that PGE2 mediates Src kinase activation and STAT3 phosphorylation in lung cancer [18]. Notably, it has been revealed that PGE2 enhanced growth hormone release in bovine anterior pituitary cells in-vitro [36]. Based on data from the current study, PGE2 expression was elevated remarkably in pituitary tumors compared with normal tissues. Moreover, an increased level of PGE2 was observed in NFPAs, invasive tumors and macro adenomas indicating the presumable role of PGE2 in pituitary tumor invasion and tumor genesis although this premise should be clarified by further studies. The simultaneous elevation of COX-2 and PGE2 in relatively similar patterns in pituitary tumors reinforces the insights regarding the consistency of COX-2 mRNA and protein in pituitary adenomas. The current study revealed no significant correlation between COX isoforms or PGE2 levels and patient’s age, gender, or tumor size which can be characterized by more populated studies although no correlation with patient’s age, tumor size, or invasiveness was observed in previous studies [31]. Notably, a large number of non-secreting pituitary adenomas that can be clinically diagnosed have macroadenoma and quite heterogenic tumors which cause pituitary insufficiency and complicate diagnosis and treatment [26]. Rationally, the presence of larger tumors in patients with NFPAs might be due to the accelerated cell proliferation and reduced cell apoptosis which facilitate tumor growth and invasion. In support of this, the apoptotic index was shown to be remarkably lower in NFPAs compared with FPAs. Moreover, the reduced level of p27 KIP1, the cyclin- dependent kinase inhibitor which suppresses the cell cycle, was observed more in NFPAs and pituitary carcinomas [37–39]. Based on the current data, higher expression levels of COX-2 isoforms and PGE2 were observed remarkably more in NFPAs compared with other types of pituitary tumors. In the case of COX-2, these elevated levels were correlated with tumor invasion. Due to the multiple lines of evidences COX-2 and PGE2 have accelerating role in cell proliferation and invasion [4] in addition NFPAs have higher rates of cell proliferation compared with other pituitary adenomas. Therefore, the up-regulation of COX-2 and PGE2 in the current study might be due to the stimulatory role of COX-2 in cell proliferation in NFPAs. Based on the fact that tumor mass is a heterogeneous population of cells, a question might be raised about the types of cells in pituitary tumors, which causes the over-production of COX pathway mediators. However, the current study evaluated equal amounts of tumor mass from different pituitary tumors and compared them so as to uncover the existence and patterns of COX isoforms expression. The type of cell that might be involved in COX isoform expression cannot be determined from the current study and should be taken in to consideration in future studies. Because of the pro-tumor genesis role of COX isoforms and PGE2 in several tumor types, the results of the current study provide new insight into the probable effect of the COX pathway in pituitary adenomas and its potential ability to be considered as an effective target in pituitary adenoma diagnosis. However, to reach a solid conclusion, evaluating the COX isoforms protein levels, enzyme activity, and the roles of other prostaglandins seems to be a prerequisite to delineate the balance between different mediators of the pathway.

## Conclusions

The current study is the first to provide data regarding the evaluation of the main COX pathway enzymes (COX-1 and COX-2) and product (PGE2) in relatively most prevalent types of pituitary adenomas, including hormone-secreting and non-secreting tumors. Accordingly, the over-expression of both enzyme isoforms was observed in tumor tissue and was more significant for COX-2 and consistent with PGE2 elevation. Among pituitary tumors, non-secreting ones revealed more expression of COX isoforms, possibly because of the higher rate of cellular proliferation in such tumors. Overall, the expression levels of COX isoforms and PGE2 levels were increased in various types of pituitary adenomas and can be considered as possible diagnostic biomarkers, especially for invasive pituitary adenomas in future surveys.

## Data Availability

All data generated or analyzed during this study and supporting our findings are included and can be found in the manuscript. The raw data can be provided by corresponding author on reasonable request.

## Abbreviations

AUC: Area under the curve
COX-1: Cycloxygensae-1
COX-2: Cycloxygensae-2
EMT: Epithelial-mesenchymal transition
ETSS: Endoscopic transnasal transphenoidal surgery
FPA: Functional pituitary adenoma
HIF2α: Hypoxia inducible factor 2α
HPA: Hypothalamic-pituitary-adrenal
MAP kinase: Mitogen-activated protein kinase
NFPA: Non-functional pituitary adenomas
NSAIDs: Nonsteroidal anti-inflammatory drug
PGE2: Prostaglandin E2
ROC: Receiver operating characteristic
ROS: Reactive oxygen species
Sp1: Specificity protein 1
STAT: Signal transducer and activator of transcription
